# Proliferation inhibition and the underlying molecular mechanisms of microRNA-30d in renal carcinoma cells

**DOI:** 10.3892/ol.2013.1754

**Published:** 2013-12-11

**Authors:** HONGSHENG YU, XIALU LIN, FANG WANG, BURONG ZHANG, WEIHUA WANG, HONGBO SHI, BAOBO ZOU, JINSHUN ZHAO

**Affiliations:** 1 Medical School, Ningbo University, Zhejiang Provincial Key Laboratory of Pathological and Physiological Technology, Ningbo, Zhejiang 315211, P.R. China; 2 Affiliated Hospital, Ningbo University, Zhejiang Provincial Key Laboratory of Pathological and Physiological Technology, Ningbo, Zhejiang 315211, P.R. China

**Keywords:** microRNA-30d, proliferation inhibition, cyclin E2, renal cell carcinoma, renal carcinoma cells

## Abstract

To investigate the inhibitory effects of microRNA-30d (miR-30d) on renal carcinoma cell proliferation and the underlying molecular mechanisms, miR-30d expression in renal cell carcinoma (RCC) specimens was analyzed by quantitative polymerase chain reaction (qPCR). The inhibition of the proliferation of miR-30d on renal carcinoma cells (ACHN cell line) was analyzed by MTT and colony formation assays. The effects of miR-30d on cyclin E2 expression were detected by the luciferase activity of the reporter gene. In addition, the effects of miR-30d on endogenous cyclin E2 expression at the RNA and protein levels were investigated by qPCR and western blot analysis, respectively. Cell cycles were analyzed by flow cytometry. The results showed the following: i) Expression of miR-30d was significantly downregulated in renal carcinoma tissues compared with paraneoplastic tissues; ii) overexpression of miR-30d inhibited renal carcinoma cell proliferation and colony formation; iii) miR-30d inhibited cyclin E2 3′ untranslated region-mediated reporter gene expression; and iv) overexpression of miR-30d downregulated endogenous cyclin E2 expression and blocked the cell cycle at the G_1_ phase. In conclusion, miR-30d functions as a tumor suppressor gene in RCC and inhibits renal carcinoma cell proliferation. Cell cycle regulatory factor cyclin E2 is a target gene of miR-30d. miR-30d inhibits renal carcinoma cell proliferation via the regulation of cyclin E2 expression at the post-transcriptional level.

## Introduction

Renal cell carcinoma (RCC) is the most common type of kidney cancer in adults, responsible for ~80% of cases (1). RCC originates from proximal tubule epithelial cells. Clear cell renal cell carcinoma (CCRCC) is the most common subtype of RCC, consisting of 70–80% of metastatic RCC (2). To date, no sensitive and specific diagnostic methods have been identified for RCC in clinical practice. In addition, RCC is relatively resistant to radiation therapy and chemotherapy (3), which results in a poor prognosis and low 5-year survival rates among patients with RCC (4). Therefore, future studies on its pathogenesis are important for the diagnosis and treatment of RCC.

Previous studies have shown that the von Hippel-Lindau (VHL) tumor suppressor gene is pivotal in RCC (5,6). Inactivating mutations of the VHL gene are a hallmark of CCRCC, since ~60% of CCRCC patients have a mutated or inactivated VHL gene (7). The wild-type protein encoded by the VHL gene inhibits the activity of the tumor proliferation proteins of β-catenin and hypoxia-inducible factor 1 (HIF-1) (5–8). The β-catenin-mediated Wnt signaling pathway and the HIF-1-activated Ras-Raf-mitogen-activated protein kinase-extracellular signal-regulated kinase signaling pathway are associated with cell proliferation (9). In addition, HIF-1 upregulates the expression of VEGF and thus, promotes angiogenesis in the tumor tissue. Therefore, inactivation of the VHL gene may indirectly relieve the inhibition of the VHL gene on β-catenin and HIF-1 proteins and thereby promote cell carcinogenesis. MicroRNAs (miRNAs/miRs) are a class of small non-coding RNA molecules. Previous studies have demonstrated the involvement of miRNAs in a wide variety of regulatory pathways, including development, virus defense, hematopoiesis organogenesis, cell proliferation, apoptosis and carcinogenesis (10,11). Compelling evidence has shown that miRNAs may also be involved in cancer initiation and progression (12,13). Mature miRNAs are generated via a two-step processing pathway to yield a 21–25-nucleotide non-coding RNA molecule that regulates gene expression (14). Mammalian miRNAs are usually complementary to a site in the 3′ untranslated region (3′ UTR) (15). miR-30 forms an miRNA family that includes miR-30a, b, c, d and e, and that has been identified in humans. Previous observations have shown that miR-30 is involved in various biological and pathological processes (16); for example, miR-30a is required for biliary morphogenesis (17), whereas miR-30c plays a role in polycythemia vera (18) and cancer cell drug resistance (19). miR-30d is localized to chromosomal region 8q24 (20). Previously, a study by Tang *et al* (21) reported that miR-30d may increase glucose-induced insulin gene transcription, but not the secretion of insulin. Furthermore, the deregulation of miR-30d in chronic lymphocytic leukemia cells (22) and anaplastic thyroid carcinoma (23) has also been observed. Similar to the VHL gene, we hypothesized that miR-30d and its inactivation or deregulation may also be important in the pathogenesis of RCC.

## Materials and methods

### Materials

The renal cancer cells (ACHN cell line) (24) and pcDNA3.1-pre-miR-30d vector were provided by the Department of Biochemistry and Molecular Biology of Peking University Health Science Center (Beijing, China). Dulbecco’s modified Eagle’s medium (DMEM) and fetal bovine serum were purchased from Gibco (Hangzhou, China), and Lipofectamine 2000 and TRIzol were obtained from Invitrogen Life Technologies (Carlsbad, CA, USA). Rabbit anti-cyclin E2 was purchased from Santa Cruz Biotechnology, Inc. (Santa Cruz, CA, USA). The pMIR-REPORT luciferase vector TaqMan miRNA assay and mirVana qRT-PCR miRNA detection kits were purchased from Ambion, Inc. (Shanghai, China). A Roche Light Cycler 480 machine (Roche Diagnostics, Indianapolis, IN, USA) was used for quantitative polymerase chain reaction (qPCR). Human RCC specimens were collected following the approval of histological detection by the Ethics Committee of the Medical School of Ningbo University (Ningbo, China). All 12 patients provided written informed consent.

### Cell proliferation and colony formation determination

A colorimetric assay using the tetrazolium salt, MTT (Sigma-Aldrich, St. Louis, MO, USA), was used to assess cell proliferation. Equivalent cell numbers (5×10^3^ cells/well) were cultured in 0.2 ml DMEM in each well. Following 1, 2, 3, 4 or 5 days of culture, 20 μl MTT (5 mg/ml) was added to each well, followed by incubation at 37°C for 3 h. Next, 150 μl dimethyl sulfoxide was added to solubilize crystals for 10 min. Plates were immediately read at 540 nm using a microplate reader (KHB ST360; Shanghai Kehua Bio-engineering Co., Ltd., Shanghai, China). Cell proliferation curves were obtained using culture days as the abscissa and MTT absorbance as the ordinate.

For the cell clone formation assay, ~1×10^3^ cells were seeded in a 35-mm cell culture dish and cultured at 37°C in a 5% CO_2_ incubator (Thermo Fisher Scientific, Rockford, IL, USA) for 14 days (until clones were visible to the naked eye). Following washing with phosphate-buffered saline (PBS), 1 ml methanol per dish was added to fix the cells for 15 min. Following staining with crystal violet dye, cell clone formation was checked under a light microscope (CKX41; Olympus, Tokyo, Japan).

### Cell cycle detection

The cells were seeded in a six-well plate. At 75–80% confluence, the cells were washed twice with PBS. Following resuspension in 0.5 ml PBS, the cells were fixed with 4.5 ml ethanol (70%) overnight. Following centrifugation (200 × g for 10 min), the cells were incubated in a PBS solution containing 0.2 mg/ml propidium iodide, 0.1% Triton X-100 and 0.1 mg/ml RNase at room temperature, avoiding light for 30 min. Flow cytometry (BD Biosciences, San Diego, CA, USA) was used to detect the cell cycle. Cell cycle distribution was analyzed using the ModFit 3.0 program (Verity Software House, Topsham, ME, USA).

### Vector construction

The 3′ UTR region of the cyclin E2 gene was amplified by qPCR using the total RNA extracted from the ACHN cells. The following PCR primers were designed: Upstream, 5′-AGA AGA TAA CTA AGC AAA CAA G-3′; and downstream, 5′-AAT GGG CTA AAA ATA AAC AGT A-3′. The PCR products were then cloned into the T-vector. Following confirmation of the target clone sequence by DNA sequencing, the clone sequence was subcloned into the report pMIR vector to form a recombinant vector named pMIR3’UTR. For the mutated vector, the QuikChange II XL site-directed mutagenesis kit (Stratagene, La Jolla, CA, USA) was used to mutate the seed sequence on the binding sites for the miR-30d cyclin E2 3′ UTR (wild-type, 5-TGTTTAC-3; mutant, 5-TGCCCTC-3; underlined sections indicate the mutated nucleotides). The constructed mutants were named pMIR3’UTRm1 and pMIR3’UTRm2, respectively.

### Plasmid transfection

The cells were seeded and cultured in a 35-mm culture dish in a 5% CO_2_ incubator at 37°C until 50% cell confluence was reached prior to transfection. A DNA mixture was prepared from varying DNA vectors (10 μg pMIR3’UTR, pMIR3’UTRm1, pMIR3’UTRm2 or pcDNA-miR30d), plus an equal volume of Opti-MEM to obtain a final volume of 2.5 ml. The DNA mixtures were added to centrifuge tubes and incubated at room temperature for 5 min. A liposome solution containing 0.1 ml Lipofectamine 2000 plus 2.4 ml Opti-MEM was prepared and incubated for 5 min. The DNA-reagent complex was prepared by diluting the DNA mixture with the liposome solution (1:1 ratio), which was then incubated for 20 min at room temperature. Next, the DNA-reagent complex was added into the ACHN cell culture medium. Following 8 h of incubation, culture medium containing the DNA-reagent complex was discarded and the cells were cultured in normal fresh medium. To obtain stably transfected cell lines, G418 was used to treat the cells for four days to select the pcDNA-miR30d clones. For the reporting measurement, the cells were further cultured for 36 h for the determination of luciferase activity. Luciferase activity in the pRL-CMV-transfected cells was used to confirm the transfection efficiency.

### qPCR

Total cellular RNA was extracted following the TRIzol method and reverse transcribed into cDNA. β-actin was used as an internal reference. The PCR primers for amplification were as follows: Cyclin E2 upstream, 5-GCA TTA TGA CAC CAC CGA AGA-3 and downstream, 5-GGC AAT CAA TCA CAG CAC TAC TT-3; and β-actin upstream, 5′-AGC GAG CAT CCC CCA AAG TT-3′ and downstream, 5′-GGG CAC GAA GGC TCA TCA TT-3′. For miR-30d qPCR expression analysis, the TaqMan miRNA assay and mirVana qRT-PCR miRNA detection kit were used. U6 small nuclear RNA was used as an internal reference, which included the following primers: Upstream, 5′-CTCGCTTCGGCAGCACA-3′ and downstream, 5′-AACGCTTCACGAATTTGCGT-3′.

### Western blot analysis

Cell lysates (30 μg protein per sample) were diluted with SDS-PAGE protein sample buffer and then incubated in a 95°C water bath for 5 min to denature the protein. SDS-polyacrylamide gel (8%) was used for the electrophoresis. Next, the proteins were electrically transferred onto a polyvinylidene fluoride (PVDF) membrane. Subsequent to being washed twice with Tris-buffered saline with Tween 20 (TBST) buffer, the PVDF membrane was blocked with 5% skimmed milk in phosphate-buffered saline with Tween 20 (PBST) buffer for 2 h. Next, the membrane was incubated with primary and secondary antibodies in 5% skimmed milk in PBST buffer for 2 h successively, washing four times with PBST buffer following incubation with each antibody. The membrane was developed in chemiluminescence reagent. Experiments were performed three times and β-actin expression was used for the control of equal protein loading.

### Statistical analysis

The intergroup differences were tested by Student’s t-test. P≤0.05 was considered to indicate a statistically significant difference.

## Results

### Expression of miR-30d in the RCC specimens

**Figure 1:**
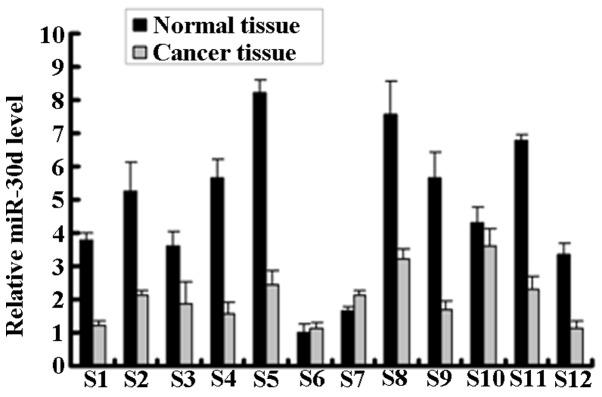
miR-30d expression in 12 renal carcinoma tissues and paired adjacent normal tissues detected by quantitative polymerase chain reaction (qPCR). miR-30d expression was significantly downregulated in the cancer tissues of 9 of the 12 renal cell carcinoma (RCC) patients. ^*^P≤0.05, vs. normal tissue. S, sample; miR-30d, microRNA-30d.

qPCR analysis was used to detect the miR-30d expression in the renal carcinoma tissue (cancer tissue) and paired adjacent normal tissue (normal tissue) samples from 12 RCC patients (Fig. 1). miR-30d expression was significantly downregulated in the cancer tissues of 9 of the 12 RCC patients.

### Proliferation inhibition of miR-30d on renal carcinoma cells

**Figure 2:**
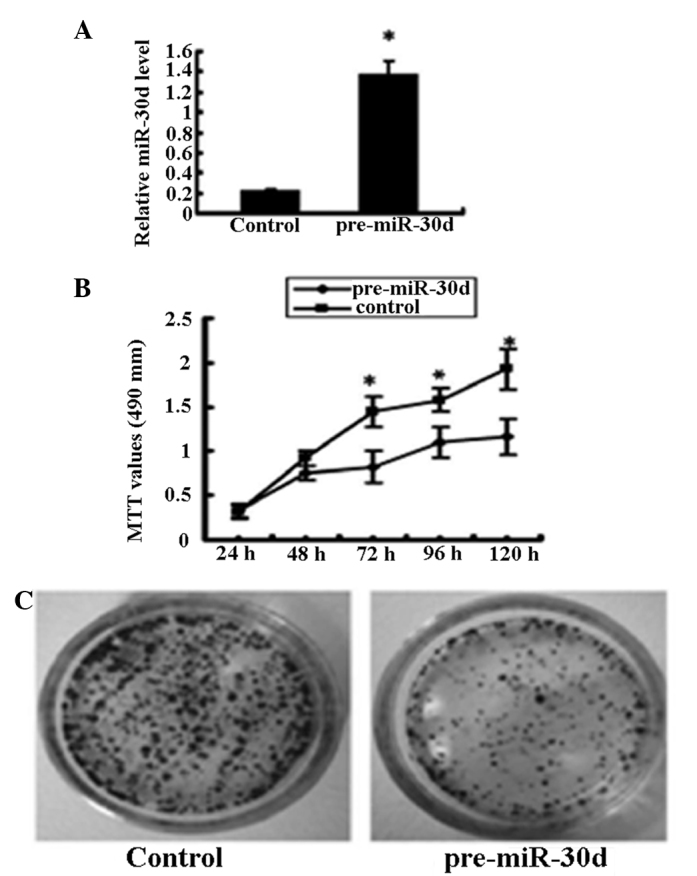
Proliferation inhibition of miR-30d in renal carcinoma cells. (A) miR-30d expression in pcDNA3.1-pre-miR-30d-transfected renal carcinoma cells was detected by quantitative polymerase chain reaction (qPCR) analysis. (B) Comparison of cell proliferation rates between pcDNA3.1-pre-miR-30d-transfected and control cells, as detected by MTT assay. (C) Comparison of cell colony formation between pcDNA3.1-pre-miR-30d-transfected and control cells. ^*^P≤0.05, vs. control. miR-30d, microRNA-30d.

To test the effects of miR-30d on cell proliferation, renal carcinoma cells (ACHN cell line) that were untransfected or stably transfected with recombinant plasmid pcDNA3.1-pre-miR-30d were studied. miR-30d expression in the pcDNA3.1-pre-miR-30d-transfected cells was upregulated six times as high as that in the untransfected cells (Fig. 2A). MTT assay showed that overexpression of miR-30d significantly inhibited renal carcinoma cell growth (Fig. 2B) and colony formation (Fig. 2C).

### Effects of miR-30d on cyclin E2 at the post-transcriptional level

**Figure 3:**
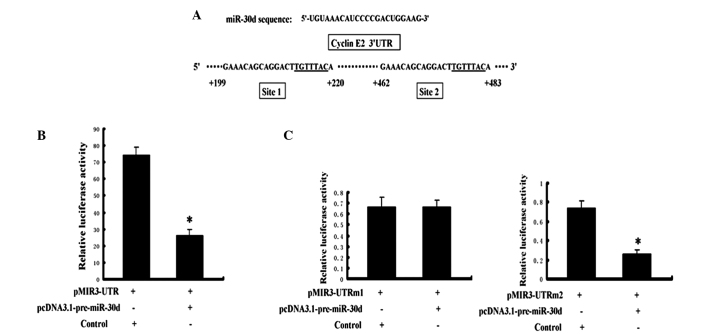
Effect of miR-30d on luciferase activity of cyclin E2 expression. (A) The miR-30d targeting sites in the cyclin E2 3′ UTR. Effect of miR-30d on the luciferase activity from (B) wild-type and (C) mutant cyclin E2 3′ UTR reporter vectors. ^*^P≤0.05 vs. control. miR-30d, microRNA-30d; UTR, untranslated region.

The cyclin E2 gene 3′ UTR reporter vectors, pMIR3’UTR and pcDNA3.1-pre-miR-30d, were co-transfected into the renal carcinoma cells. To further confirm that miR-30d directly regulates cyclin E2, the cyclin E2 3′ UTR and miR-30d were mutated on the seed sequence of the binding sites. Fig. 3A presents the two miR-30d binding sites on the cyclin E2 3′ UTR (named site 1 and site 2; underlined section indicates the seed sequence). The activity of the reporter gene luciferase was significantly inhibited by miR-30d (Fig. 3B), and the mutation at site 1 significantly attenuated the inhibition of miR-30d (Fig. 3C, left). However, the mutation at site 2 exhibited no effect on this inhibition (Fig. 3C, right).

**Figure 4:**
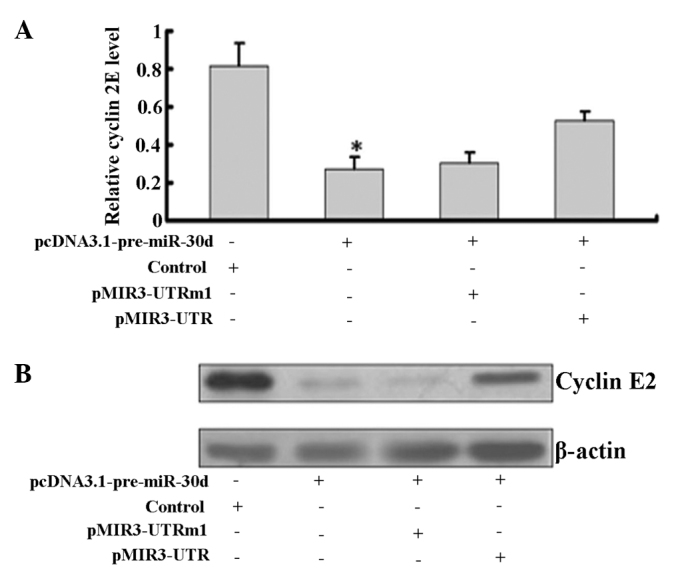
Effect of miR-30d on endogenous cyclin E2 expression in renal carcinoma cells. Effect of miR-30d on endogenous cyclin E2 at the (A) mRNA level, as detected by quantitative polymerase chain reaction (qPCR) analysis, and at the (B) protein level, as detected by western blot analysis, in renal carcinoma cells. ^*^P≤0.05 vs. control. miR-30d, microRNA-30d; UTR, untranslated region.

Western blot analysis showed that overexpression of miR-30d significantly downregulated cyclin E2 at the mRNA and protein levels in pcDNA3.1-pre-miR-30d-transfected cells (Fig. 4).

### Effects of miR-30d on the renal carcinoma cell cycle

**Figure 5:**
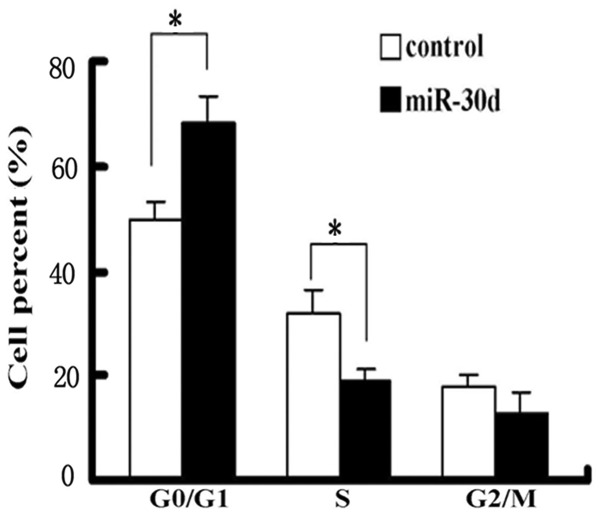
Effect of miR-30d on the renal carcinoma cell cycle. miR-30d expression significantly increased the content of the cells in the G_1_ phase and decreased the content of the cells in the S phase. ^*^P≤0.05 vs. control. miR-30d, microRNA-30d.

Renal carcinoma cells were collected 48 h after transfection with pcDNA3.1-pre-miR-30d. The cell cycle was analyzed using flow cytometry. Compared with the control group, miR-30d expression significantly increased the content of the cells in the G_1_ phase and simultaneously decreased S-phase cell percentages (Fig. 5).

## Discussion

Kidney cancer is one type of urinary system tumor with a high degree of malignancy, and its pathogenesis remains unclear. Previous studies have demonstrated that miRNA may be associated with the occurrence and development of kidney cancer (25–27). Studies have demonstrated that the expression of miR-185 in RCC is significantly upregulated (28). miR-185 inhibits the expression of the tumor suppressor gene, PTEN. PTEN is an important inhibitor of the phosphoinositide 3-kinase (PI3K)/Akt pathway, and its downregulation may lead to activation of this signaling pathway (28,29). An additional study showed that the miRNA expression of miR-26a, -221/222, -199a-5p, -449a, -21 and -34a may be associated with RCC (30).

The present study showed that the expression of miR-30d in renal carcinoma tissue was significantly lower than in the paired adjacent normal tissue. Conversely, miR-30d overexpression significantly inhibited the proliferation and colony formation of renal carcinoma cells in culture, indicating that miR-30d has the function of a tumor suppressor. Consistent with this feature, it was further demonstrated that miR-30d binds to cyclin E2 and inhibits its expression. To confirm this prediction, the cyclin E2 gene 3′ UTR reporter vectors, pMIR3’UTR and pcDNA3.1-pre-miR-30d, were co-transfected into the renal carcinoma cells. The results indicated that the reporter gene luciferase activity was significantly inhibited by miR-30d, confirming that cyclin E2 is a target gene of miR-30d. Further experiments indicated that miR-30d regulates the expression of cyclin E2 by binding to the 3′ UTR. To test this hypothesis, the cyclin E2 3′ UTR and miR-30d were mutated on the seed sequence of the binding sites. The results indicated that site 1 mutations attenuate the inhibition of miR-30d, however, site 2 mutations exhibited no such inhibitory effect, suggesting that miR-30d regulates the expression of cyclin E2 by combining with site 1. Further experiments indicated that the overexpression of miR-30d significantly downregulates cyclin E2 at the mRNA and protein levels in pcDNA3.1-pre-miR-30d-transfected cells. In addition, overexpression of miR-30d may block renal carcinoma cells at the G_1_ phase, suggesting that cell cycle arrest is an important mechanism for miR-30d inhibition on the RCC cell proliferation. Cyclin E2 is a positively regulated factor in the G_1_ phase of the cell cycle. The cyclin E2 cyclin-dependent kinase 2, composed of the serine/threonine kinase complex, regulates the G_1_ phase of the process. These results indicated that miR-30d inhibits the expression of cyclin E2, which results in renal carcinoma cell cycle arrest.

Previously, Esposito *et al* (31) reported that the downregulation of miR-30d is associated with the incidence of thyroid cancer. In addition, Chen *et al* (32) found that the overexpression of miR-30d inhibits the proliferation of HeLa cells. miRNA expression profiling for chronic lymphocytic leukemia also showed downregulated miR-30d in leukemia cells (22). These observations are consistent with the results of the current study, indicating that miR-30d acts as a tumor suppressor gene. However, certain other studies have previously indicated that miR-30d may also act in a similar manner to the oncogenes, since the upregulation of miR-30d has been reported in hepatocellular carcinoma (16). Kumar *et al* (33) previously found that miR-30d inhibits the expression of p53. In addition, the overexpression of miR-30d genes has been found in medulloblastoma cells (34). These disparities in results may be due to the difference in cell types, indicating that the exact roles of miR-30d (acting as an oncogene or tumor suppressor) may be varied in different cells. Therefore, future studies are necessary to elucidate the precise molecular mechanism of miR-30d in the occurrence and development of various types of cancer.

In conclusion, the results of the current study indicated that miR-30d is important in the regulation of RCC proliferation. The downregulation of miR-30d may be associated with the occurrence and development of RCC. Due to the inhibitory effects of miR-30d on the proliferation of renal carcinoma cells, this may be a useful new target for the diagnosis and treatment of kidney cancer.

## References

[1] Farber LJ, Furge K, Teh BT (2012). Renal cell carcinoma deep sequencing: recent developments. Curr Oncol Rep.

[2] Reuter VE (2006). The pathology of renal epithelial neoplasms. Semin Oncol.

[3] Itsumi M, Tatsugami K (2010). Immunotherapy for renal cell carcinoma. Clin Dev Immunol.

[4] Walsh N, Larkin A, Kennedy S (2009). Expression of multidrug resistance markers ABCB1 (MDR-1/P-gp) and ABCC1 (MRP-1) in renal cell carcinoma. BMC Urol.

[5] Linehan WM, Rubin JS, Bottaro DP (2009). VHL loss of function and its impact on oncogenic signaling networks in clear cell renal cell carcinoma. Int J Biochem Cell Biol.

[6] Linehan WM, Pinto PA, Srinivasan R (2007). Identification of the genes for kidney cancer: opportunity for disease-specific targeted therapeutics. Clin Cancer Res.

[7] An J, Rettig MB (2005). Mechanism of von Hippel-Lindau protein-mediated suppression of nuclear factor kappa B activity. Mol Cell Biol.

[8] Peruzzi B, Athauda G, Bottaro DP (2006). The von Hippel-Lindau tumor suppressor gene product represses oncogenic β-catenin signaling in renal carcinoma cells. Proc Natl Acad Sci USA.

[9] Conrad PW, Freeman TL, Beitner-Johnson D, Millhorn DE (1999). EPAS1 trans-activation during hypoxia requires p42/p44 MAPK. J Biol Chem.

[10] Carrington JC, Ambros V (2003). Role of microRNAs in plant and animal development. Science.

[11] Yanaihara N, Caplen N, Bowman E (2006). Unique microRNA molecular profiles in lung cancer diagnosis and prognosis. Cancer Cell.

[12] Bartel DP (2009). MicroRNAs: target recognition and regulatory functions. Cell.

[13] Horikawa Y, Wood CG, Yang H (2008). Single nucleotide polymorphisms of microRNA machinery genes modify the risk of renal cell carcinoma. Clin Cancer Res.

[14] Trang P, Weidhaas J, Slack F (2008). MicroRNAs as potential cancer therapeutics. Oncogene.

[15] Didiano D, Hobert O (2006). Perfect seed pairing is not a generally reliable predictor for miRNA-target interactions. Nat Struct Mol Biol.

[16] Yao J, Liang L, Huang S (2010). MicroRNA-30d promotes tumor invasion and metastasis by targeting Galphai2 in hepatocellular carcinoma. Hepatology.

[17] Hand NJ, Master ZR, Eauclaire SF, Weinblatt DE, Matthews RP, Friedman JR (2009). The microRNA-30 family is required for vertebrate hepatobiliary development. Gastroenterology.

[18] Bruchova H, Merkerova M, Prchal JT (2008). Aberrant expression of microRNA in polycythemia vera. Haematologica.

[19] Sorrentino A, Liu CG, Addario A, Peschle C, Scambia G, Ferlini C (2008). Role of microRNAs in drug-resistant ovarian cancer cells. Gynecol Oncol.

[20] Schlaeger C, Longerich T, Schiller C (2008). Etiology-dependent molecular mechanisms in human hepatocarcinogenesis. Hepatology.

[21] Tang X, Muniappan L, Tang G, Ozcan S (2009). Identification of glucose-regulated miRNAs from pancreatic (beta) cells reveals a role for miR-30d in insulin transcription. RNA.

[22] Marton S, Garcia MR, Robello C (2008). Small RNAs analysis in CLL reveals a deregulation of miRNA expression and novel miRNA candidates of putative relevance in CLL pathogenesis. Leukemia.

[23] Visone R, Pallante P, Vecchione A (2007). Specific microRNAs are downregulated in human thyroid anaplastic carcinomas. Oncogene.

[24] Wu C, Jin B, Chen L (2013). MiR-30d induces apoptosis and is regulated by the Akt/FOXO pathway in renal cell carcinoma. Cell Signal.

[25] Petillo D, Kort EJ, Anema J, Furge KA, Yang XJ, Teh BT (2009). MicroRNA profiling of human kidney cancer subtypes. Int J Oncol.

[26] Nakada C, Matsuura K, Tsukamoto Y (2008). Genome-wide microRNA expression profiling in renal cell carcinoma: significant down-regulation of miR-141 and miR-200c. J Pathol.

[27] Juan D, Alexe G, Antes T (2010). Identification of a microRNA panel for clear-cell kidney cancer. Urology.

[28] Pantuck AJ, Seligson DB, Klatte T (2007). Prognostic relevance of the mTOR pathway in renal cell carcinoma. Cancer.

[29] Rathmell WK, Chen S (2008). VHL inactivation in renal cell carcinoma: implications for diagnosis, prognosis and treatment. Expert Rev Anticancer Ther.

[30] Redova M, Svoboda M, Slaby O (2011). MicroRNAs and their target gene networks in renal cell carcinoma. Biochem Biophys Res Commun.

[31] Esposito F, Tornincasa M, Pallante P (2012). Down-regulation of the miR-25 and miR-30d contributes to the development of anaplastic thyroid carcinoma targeting the polycomb protein EZH2. J Clin Endocrinol Metab.

[32] Chen LZ, Niu XH, Han XL, Xie BS, Mao ZB (2009). Down-regulation of miR-30d on proliferation of HeLa cells. Chinese Journal of Biochemistry and Molecular Biology.

[33] Kumar M, Lu Z, Takwi AA (2010). Negative regulation of the tumor suppressor p53 gene by microRNAs. Oncogene.

[34] Lu Y, Ryan SL, Elliott DJ (2009). Amplification and overexpression of Hsa-miR-30b, Hsa-miR-30d and KHDRBS3 at 8q24.22–q24.23 in medulloblastoma. PLoS One.

